# Transient Voltage Information Entropy Difference Unit Protection Based on Fault Condition Attribute Fusion

**DOI:** 10.3390/e27010061

**Published:** 2025-01-11

**Authors:** Zhenwei Guo, Ruiqiang Zhao, Zebo Huang, Yongyan Jiang, Haojie Li, Yingcai Deng

**Affiliations:** School of Mechanical and Electrical Engineering, Guilin University of Electronic Technology, Guilin 541004, China; zwguoxy@guet.edu.cn (Z.G.); iamjyyan@163.com (Y.J.); lihj327@163.com (H.L.); d18378392337@163.com (Y.D.)

**Keywords:** transient voltage unit protection, fault condition properties, fusion of fault information, variational modal decomposition (VMD), information entropy

## Abstract

Transient protection has the advantage of ultra-high-speed action, but traditional transient protection is susceptible to the influence of two fault condition attributes, namely, transition resistance and initial angle of fault, and there are the problems of insufficient sensitivity and insufficient reliability under weak faults. To this end, the propagation characteristics of high-frequency components of transient voltage in bus and line systems are explored, and a new method of unit protection based on the entropy difference in transient voltage information is proposed. In order to solve the problem of single-ended transient protection not being able to reliably distinguish line faults from bus faults and adjacent line first-end faults, the difference between the entropy of line voltage and the entropy of bus voltage was introduced as a fault characteristic. Aimed at the susceptibility of transient protection to the influence of fault condition attributes, composite fault characteristics containing fault attribute information were obtained by integrating fault characteristics with fault condition attributes to overcome the adverse influence of fault condition attributes on transient protection and improve the reliability of the protection. The algorithm solved 38.9% of the original cross-data, 36.1% of the false actions, and 6.1% of the rejected actions. Finally, the accuracy and reliability of the proposed algorithm were verified by extensive ATP-Draw simulation tests.

## 1. Introduction

Due to the inverse distribution characteristics of primary energy and power loads, ultra-high-voltage (UHV) and extra-high-voltage transmission technologies with large capacity and long distances are widely used [1]. To increase the stability and dependability of power systems, it is increasingly important to provide faster protection methods [2]. Fault-induced transient information contains rich fault details, including fault form, fault location, fault duration, and so on [3,4]. Transient protection is immune from network fluctuation and transformer saturation, allowing for ultra-fast fault detection and localization in as little as 10 ms [5]. Therefore, protection schemes based on transient quantities have received widespread attention [6,7].

The literature [8] distinguishes between within and exterior bus failures by measuring the proportional size of the initial traveling wave reactive power of all lines correlated to the bus. Reference [9] analyzes the main frequency of grid transient quantities and the distinction between energy levels at high and low frequencies of the interior and exterior faults in the guarded section and constructs the main frequency range energy qualities. The main frequency feature of the transient differential current is retrieved using the least squares fitting approach to complete longitudinal protection [10]. Reference [11] uses the ratio of forward and reverse traveling wave amplitudes to construct an in- and out-of-zone fault identification method. Reference [12] uses the initial traveling wave arrival time at both ends of the transmission line to achieve fast protection. In [13], a new method of transient protection based on the principle of directional comparison of transient energy is proposed. Variational modal decomposition (VMD) can decompose the signal into single-component intrinsic modal function components, which is conducive to improving the time-domain and frequency-domain resolution of fault signals [14]. Some studies [15] used VMD to break the fault voltage signal down into distinct frequency range signals, and used conjugate gradient frequency division to invert each component to achieve accurate detection of voltage traveling waves. Reference [16] extracted five kinds of time–frequency eigen quantities to construct a fault transient information fusion matrix and used the difference and similarity of the matrix to achieve single-ended fault localization. The traps that are placed in the terminals of power lines have the property of hindering the propagation of transient high-frequency signals through them, while hardly affecting the mid-range and low frequencies [17]. Before and after a faulty high-frequency signal passes through a trap, the difference in fault characteristics is obvious. Some studies [18] have analyzed the propagation characteristics at the line boundary of the trap and the busbar’s distributed capacitance.

Research on transient traveling wave protection has made encouraging progress in different fields [19]. However, these protection methods suffer from the difficulty of relatively low reliability. If the beginning angle of the fault has tiny or large transition resistance, the fault transient signal is obviously weakened, and the faults’ properties are not readily apparent [20]. It is difficult for traditional traveling wave protection to meet the requirements due to its dependence on the wave head detection of the traveling wave [21]. The difference between the fault characteristics during weak faults and those during strong faults is huge, and it is difficult to incorporate various fault conditions in action value adjustment. Reference [22] points out that within the range of 18° before and after the initial fault angle crossing the zero point, transient traveling wave directional protection is not sensitive enough, and its reliability cannot meet the requirements. Studies on transient traveling wave protection need to continue to explore new methods with better performance.

This research proposes a novel unit protection technology based on the entropy difference in instantaneous high-frequency voltage. The main contributions of this paper are as follows:

1. The impact of fault condition attributes on fault characteristics is investigated. The findings indicate that these attributes are critical factors influencing the reliability of conventional single-ended transient protection.
2. In order to overcome the adverse effects of fault attributes on transient protection, fault characteristics and fault condition attributes are fused to obtain comprehensive fault characteristics containing fault attribute information. A new instantaneous protection standard is established by employing the integrated fault characteristics.
3. Different from traditional bus protection, which needs to extract the fault information of all the lines connected to the bus, the entropy difference between the high-frequency signal of bus voltage and the high-frequency signal of the voltage of one line is introduced to construct the fault feature. The proposed algorithm, which can protect a bus and a line simultaneously, is simpler and more economical.

The remaining portions of the paper are structured as follows: Section 2 describes the information entropy calculation method for faulty high-frequency voltage signals. The suggested algorithm’s essential idea and operation are explained in Section 3. Section 4 verifies the correctness of the approach through extensive simulations. Section 5 summarizes the features and advantages of the proposed protection method.

## 2. Preliminaries

This section introduces the concept of the VMD technique as well as its iterative solution process and the calculation method of high-frequency information entropy.

### 2.1. VMD of Fault Voltage Signal

The VMD approach effectively eliminates the endpoint impact and modal part mixing issues inherent in empirical modal decomposition. It also reduces the non-smoothness of the time series characterized by significant nonlinearity and complexity, resulting in relatively smooth subseries with distinct frequency scales. The VMD algorithm demonstrates greater stability and accuracy during signal decomposition, being less prone to modal confusion and over-decomposition. This enables the extraction of cleaner high-frequency components and mitigates the influence of noise on signal decomposition and subsequent information entropy analysis. Consequently, the fault voltage signal is decomposed using the VMD to isolate the required high-frequency components. 

VMD is an adaptive, fully non-recursive, quasi-orthogonal signal processing method capable of decomposing a wide-band signal *f (ω)* into several intrinsic modal IMF components $u_{k}$, each centered around its corresponding frequency $\omega_{k}$ [23]. The iterative process of $u_{k}\left(\omega \right)$ and $\omega_{k}$ in the Fourier domain is shown in Equations (1) and (2).

(1) $${\overset{\wedge }{u}}_{k}^{n+1}\left(\omega \right)=\frac{\overset{\wedge }{f}\left(\omega \right)-\sum_{i\neq k}\overset{\wedge }{u}\left(\omega \right)+\frac{\overset{\wedge }{\lambda }\left(\omega \right)}{2}}{1+2\alpha {\left(\omega -\omega_{k}\right)}^{2}}$$

(2) $$\omega_{k}^{n+1}=\frac{\int_{0}^{\infty }\omega {\left|\overset{\wedge }{u_{k}}\left(\omega \right)\right|}^{2}d\omega }{\int_{0}^{\infty }{\left|\overset{\wedge }{u_{k}}\left(\omega \right)\right|}^{2}d\omega }$$

where ${\hat{u}}_{k}^{n+1}\left(\omega \right)$ signifies the Wiener filter of the modal signal, representing the Fourier transform of $u_{k}^{n+1}\left(t\right)$; $\omega_{k}^{n+1}$ represents the center of gravity of the power spectrum corresponding to the modal function; *λ* is the second penalty factor; and *α* is the Lagrange multiplier.

The expression for *λ* after alternating optimization iterations is

(3) $${\overset{\wedge }{\lambda }}^{n+1}\left(\omega \right)\leftarrow {\overset{\wedge }{\lambda }}^{n}\left(\omega \right)+\gamma \left(\overset{\wedge }{f}\left(\omega \right)-\sum {\overset{\wedge }{u}}_{k}^{n+1}\left(\omega \right)\right)$$

where γ is the noise tolerance, which satisfies the fidelity requirement for signal decomposition.

The following is the primary iterative resolution procedure used by VMD:

1. Initialize $u_{k}^{1}$, $\omega_{k}^{1}$, and $\lambda_{k}^{1}$ with initial values set to 0. Set the maximum number of iterations and *k* as a positive integer to be decomposed.
2. Update ${\hat{u}}_{k}$ and $\omega_{k}$ using Equations (1) and (2).
3. Update $\hat{\lambda }$ using Equation (3).
4. Set the accuracy convergence criterion as ε > 0. If $\sum_{k}{\left\|{\hat{u}}_{k}^{n+1}-{\hat{u}}_{k}^{n}\right\|}_{2}^{2}∕{\left\|{\hat{u}}_{k}^{n}\right\|}_{2}^{2}<\varepsilon$ and *n* < N are not met, then go back to Step 2; otherwise, conclude the current iteration and produce the ultimate outcomes for ${\hat{u}}_{k}$ and $\omega_{k}$.

### 2.2. Fault Voltage Information Entropy

The fault’s transitory features are readily detected in the high-frequency portion of the fault signal. According to information entropy theory, entropy can reflect the complexity and uncertainty of a system’s state, making it a useful tool for mining large datasets [24]. Consequently, the information entropy of the fault voltage traveling wave can reflect the changes before and after the fault, making it a suitable characteristic for fault detection. Therefore, after the fault voltage is decomposed using VMD, the information entropy of the high-frequency voltage component is calculated as a transient quantity that describes the fault.

Let *E* = *E*_1_, *E*_2_, …, *E_i_* represent the energy spectrum of the VMD of the signal *x*(*t*). The signal energy generates a partition of *E*. By summing all the element powers *E_i_*, the total signal power *E* in a given time interval is obtained. Let *P_i_* = *E_i_*/*E*; then,

(4) $$\sum_{i}p_{i}=1$$

In (5), *P_i_* indicates the energy proportion of each mode component.

The information entropy *VEE* of each component in the VMD of the fault voltage signal can be calculated using Equation (6).

(5) $$VEE=-\sum_{i}p_{i}\log p_{i}$$

## 3. Materials and Methods

This section describes the frequency characteristics of the line boundary, the fault characteristics based on the voltage information entropy difference, and the problems present in traditional single-ended transient protection, which are illustrated through simulation. Additionally, the proposed protection algorithm flow is presented.

### 3.1. Line Boundary Frequency Characteristics

**Figure 1:**
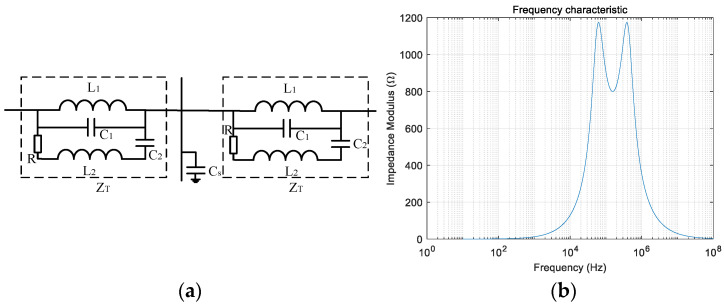
Introduction to the line trap. (**a**) Line trap configuration diagram; (**b**) impedance characteristics of line trap.

The two ends of the ultra-high-transmission line are fitted with a trap, which has the effect of preventing high-frequency signals from passing through, while medium- and low-frequency signals, such as industrial-frequency signals, propagate almost unaffected. The trap shown in Figure 1a is an XZF-3150-1.0/63-B1-type band-tuned trap with the following specific parameters: L_1_ = 1 mH, C_1_ = 3466 pF, R = 800 Ω, C_2_ = 1563 pF, L_2_ = 2.22 mH, and Z_T_ = sL_1_//(1/sC_1_) // (R + sL_2_ + 1/sC_2_). The impedance frequency characteristics of this model are shown in Figure 1b. The trap exhibits high impedance for transient signals located in the high-frequency band of [42, 580] kHz, with impedance values greater than 800 Ω, while it exhibits low impedance for signals of medium and low frequencies. The transient signals in the resistance band are significantly attenuated by the trap, which forms the line border of the erroneous high-frequency signals [17].

### 3.2. Construction of the Entropy Difference in Transient Voltage

**Figure 2:**
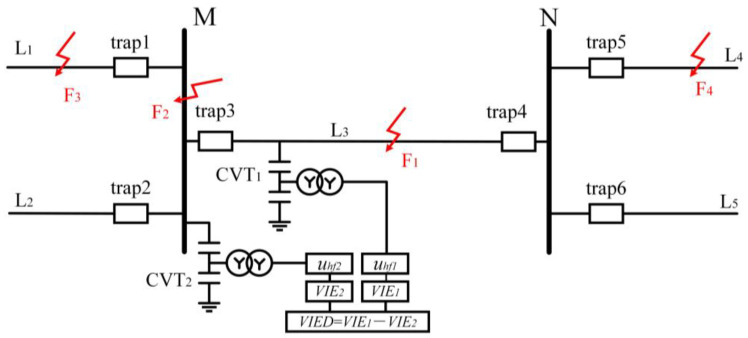
Diagram of UHV transmission system.

In the transmission system depicted in Figure 2, traps are provided at both ends of all the lines. After passing through the traps, the defective high-frequency signal experiences significant attenuation. Let the voltage detected by the line voltage transformer CVT_1_ be *u_1_*, and the voltage detected by the bus CVT_2_ be *u*_2_. The high-frequency elements of *u*_1_ and *u*_2_ are denoted as *u_hf_*_1_ and *u_hf_*_2_, respectively. In the following, we analyze the characteristics of the change in the high-frequency components of the transient voltages when malfunctions occur at the locations of F_1_, F_2_, F_3_, and F_4_, respectively. To enhance the stability of the technique, the information entropy of *u_hf_*_1_ and *u_hf_*_2_ is taken as the object of study. The information entropy of *u_hf_*_1_ and *u_hf_*_2_ are denoted as *VIE*_1_ and *VIE*_2_, respectively. The difference between the information entropy of *u_hf_*_1_ and that of *u_hf_*_2_ is defined as VIED, which is shown in Equation (7).

(6) $$VIED=VIE_{1}-VIE_{2}$$

When a fault occurs in line L_3_, the *u_hf_*_1_ detected by CVT_1_ represents the transient voltage that has not been attenuated by the traps, and its information entropy VIE1 is very large. The u2 detected by CVT2 is the instantaneous voltage after passing through trap3, and *u_hf_*_2_ is the high-frequency component obtained after a single attenuation by the traps. *VIE*_1_ is much larger than *VIE*_2_. At this point, *VIED* > 0, and *VIED* is a large positive value. When a fault occurs in line L_4_, the *u_hf_*_1_ detected by CVT_1_ is the instantaneous high-frequency voltage section after secondary attenuation by trap4 and trap5. The *u*_2_ detected by CVT_2_ is the instantaneous voltage after passing through trap5, trap4, and trap3, and *u_hf_*_2_ is the high-frequency component obtained after three-time attenuation by the traps. Both *VIE*_1_ and *VIE*_2_ are smaller, but *VIE*_1_ is still much larger than *VIE*_2_. At this point, *VIED* > 0, and *VIED* is a smaller positive value.

When the bus M is faulted, the *u_hf_*_2_ detected by CVT_2_ is the high-frequency portion of the fault transient voltage that has not been attenuated by the traps, and it has a large value of information entropy. The *u_hf_*_1_ detected by CVT_1_ is the instantaneous high-frequency voltage after one attenuation by trap3. The information entropy of *u_hf_*_2_, *VIE*_2_, is much larger than the information entropy of *u_hf_*_1_, *VIE*_1_. At this point, *VIED* < 0, and *VIED* is a negative value with a large absolute value. When the fault occurs in line L_1_, the *u_hf_*_2_ detected by CVT_2_ is the instantaneous high-frequency voltage section after one attenuation by trap1. The *u*_1_ detected by CVT_1_ is the instantaneous voltage after passing through trap1 and trap3, and *u_hf_*_1_ is the high-frequency component obtained after secondary attenuation by the traps. The information entropy *VIE*_1_ of *u_hf_*_1_ and the information entropy *VIE*_2_ of *u_hf_*_2_ are both smaller, but *VIE*_2_ is still much larger than *VIE*_1_. At this point, *VIED* < 0, and *VIED* is a negative number with a small absolute value.

From the above analysis, the following conclusions can be drawn: *VIED* is a very large positive number only when line L_3_ is faulted; *VIED* is a very large negative number in absolute value when, and only when, bus M is faulted; and *VIED* is small in absolute value when faults occur in other areas. Therefore, *VIED* may be applied to bus M and line L_3_ as a malfunction indicator.

### 3.3. The Impact of Fault Condition Attributes on Protection

Reference [22] points out that the fault eigenvalue when the initial fault angle is −5° is 10% of the amplitude when the initial phase angle is 90°. At this point, the protection sensitivity cannot meet the requirements, and reduced sensitivity of the safeguard occurs as the malfunction’s beginning angle increases. When the initial phase angle is within 18° before and after the zero point, the protection reliability does not meet the requirements. The beginning phase angle and malfunction impedance also significantly affect the difference between the information entropy of the high-frequency portion of the malfunction’s instantaneous voltage and the information entropy of the signal at high frequencies.

If the traditional method is used, *VIED* is employed simply as L_3_’s malfunction feature. The value of *VIED* is taken as shown in Table 1 for faults in the zone under different fault condition attributes. For example, when *R_f_* = 0 Ω, the *VIED* with *θ_f_* of 1° is 0.2% of the *VIED* with *θ_f_* of 90°. Similarly, the *VIED* with *θ_f_* of 5° is 3% of the *VIED* with *θ_f_* of 90°. In this case, the protection sensitivity does not meet the requirements.

**Figure 3:**
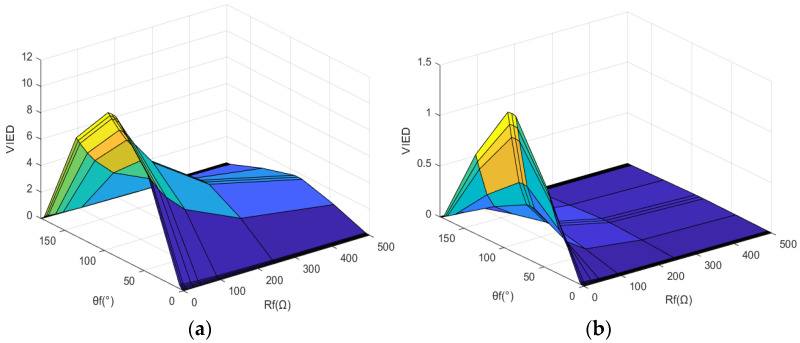
The *VIED* change curve. (**a**) Line L_3_ end fault; (**b**) bus N fault.

In addition, the fault condition attribute can lead to unreliable fault identification for line end and remote bus faults. Figure 3 displays the VIED when L_3_ and bus N are faulty under different condition attributes. There is a 38.9% overlap between the data for internal and external faults. To ensure that all internal faults on line L_3_ are detected, 36.1% of external faults on line L_4_ would result in false operations. Conversely, to prevent false operations for external faults, the rejection rate for internal faults within an 11° range around the zero-crossing point is 6.1%. Considering all fault conditions, both the false operation rate and the rejection rate will be higher. Therefore, if traditional methods are used to set protection action values directly based on these data, it will be impossible to meet the requirement of reliably detecting internal faults while ensuring reliable non-operation for external faults.

Traditionally, it has been difficult to ensure that the reliability of protection under all operating conditions meets the requirements by adjusting the action value for line L_3_ based solely on the *VIED*, without considering the role of fault condition attributes. Therefore, this thesis proposes that the method of considering fault condition attributes will solve this problem.

### 3.4. Information Entropy Differential Protection Algorithm Incorporating Fault Condition Attribute Information

In this paper, it is found that the malfunction instantaneous impedance and the beginning phase angle are two key factors affecting the value of the fault characteristic *VIED*. Therefore, the malfunction beginning phase angle and malfunction impedance are described as two fault condition attributes of the fault characteristic *VIED*. Using the method shown in Equations (8) and (9), these two fault condition attributes are fused into the fault characteristics to obtain the synthetic fault characteristics (S*VIED*).

(7) $$SVIED=\frac{VIED}{f\left(\theta_{f},R_{f}\right)}$$

(8) $$\begin{array}{cc} f\left(\theta_{f},R_{f}\right)= & \gamma \cdot \pi \left(a\cdot \exp\left(b\cdot R_{f}\right)+c\cdot \exp\left(d\cdot R_{f}\right)\right)\\ & \cdot \left(a_{0}+a_{1}\cdot \cos\left(\omega \cdot \theta_{f}\right)+b_{1}\cdot \cos\left(\omega \cdot \theta_{f}\right)\right) \end{array}$$

where *θ_f_* and *R_f_* are the fault beginning phase angle and malfunction impedance, respectively, and *a*, *b*, *c*, *d*, *a*_0_, *a*_1_, *b*_1_, and ω are given constants. These constants can be obtained from experimental typical fault data using a data fitting method.

The common methods for calculating *θ_f_* include the modal component, instantaneous power method, and phase coefficient method. In this algorithm, the phase coefficient method is used. The fault distance, the fault angle at the protection installation, and the line phase coefficient are used to calculate *θ_f_*. Reference [25] proposes a method for calculating *R_f_* using the active power detected by the protection device.

**Figure 4:**
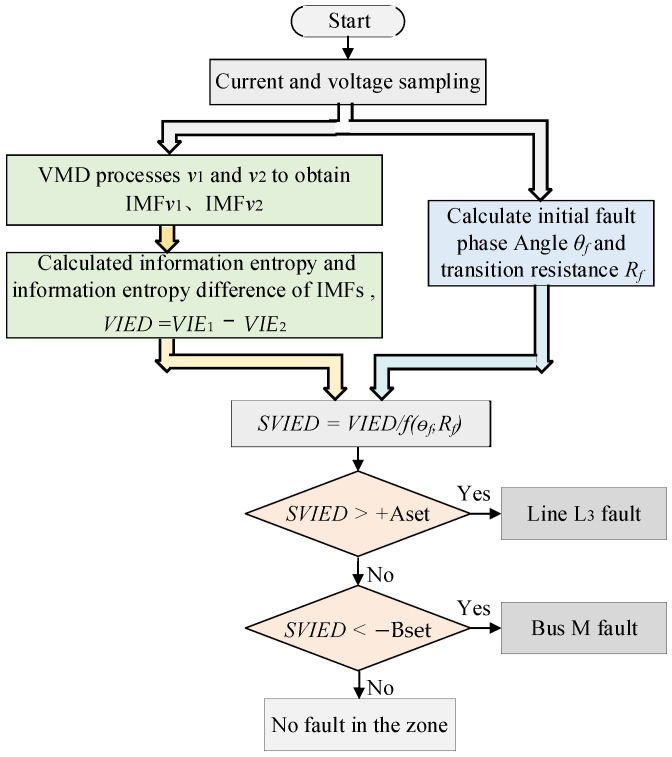
Flowchart of the protection algorithm.

*SVIED*, which incorporates two fault condition attributes, becomes a four-dimensional dataset with multiple pieces of information. In the fault judgment process, the integrated fault characteristic examines the malfunctioning characteristic quantities analyzed from the viewpoint of the malfunction condition attribute, thus overcoming the influence of the fault condition attributes on the fault characteristic value. This significantly improves the accuracy of fault judgment and enhances protection reliability. The comprehensive fault characteristics include two fault condition attributes. In the fault judgment process, the suggested protection algorithm consists of the following six steps, and the algorithm flowchart is illustrated in Figure 4. The pseudocode for the proposed algorithm is presented in Pseudocode 1.

Pseudocode 1 **Transient unit protection algorithm.**

  Initialize fault area (fa), the balancing parameter of the data-fidelity constraint (alpha), time-step of the dual ascent (tau), the number of modes to be recovered (K), true if the first mode is put and kept at DC (0-freq) (DC), all omegas start at value (init), tolerance of convergence criterion (tol).

  Begin

  evaluate the signal decomposition function *VMD *(signal, alpha, tau, K, DC, init, tol) for voltage signal *u*_i_ with *i* = 1, 2

  generate information entropy of high-frequency components using (5)

  generate difference of information entropy of high-frequency components using (6)

  Calculate fault start angle *θ_f_*

  Calculate fault transition resistance *R_f_*

  generate synthetic fault characteristics using (7)

  /* determine as Line L_3_*/

  if synthetic fault characteristics* > *a positive setting value

  update the fault area variable fa

  /* determine as bus M fault */

  else if synthetic fault characteristics* < *a negative setting value

  update the fault area variable *fa*

  /* determine as outer-zone fault */

  else

  update the fault area variable fa

  end if

  end

1. Fault voltage signals *u_1_* and *u_2_* are collected from CVT_1_ of line L_3_ and CVT_2_ of bus M, respectively.
2. VMD decomposes *v_1_* and *v_2_* to obtain their intrinsic mode functions, IMF*v*_1_ and IMF*v*_2_, respectively.
3. The information entropy values, *VIE*_1_ and *VIE*_2_, of the high-frequency IMF*v*_1_ and IMF*v*_2_ are computed, and the difference in information entropy *VIED* is calculated as *VIED,* where *VIED* = *VIE*_1_–*VIE*_2_.
4. The malfunction beginning phase angle *θ_f_* and transition resistance *R_f_* are calculated.
5. The information of *VIED* is fused with the fault condition attributes *θ_f_* and *R_f_* to obtain *SVIED*.
6. Fault judgment: if *SVIED* > +Aset, then a fault in line L_3_ is detected; otherwise, if *SVIED* < −Bset, then a fault in bus M is detected; otherwise, an out-of-area fault is identified. Aset represents the protection action threshold for line L_3_, with a value of 6, and Bset indicates the protection action threshold for bus M, with a value of 1500.

## 4. Experiments

The experimental outcomes of the suggested fault diagnostic approach for a model of a UHV transmission system are demonstrated in this section. In Section 4.1, the settings of the simulation system model are described. Section 4.2 and Section 4.3 illustrate the *SVIED* under various fault situations. Section 4.4 describes the setting of fault criterion. Experimental findings confirm that the suggested approach is successful. Section 4.5 illustrates the influence of fault location on protection, provides a qualitative data analysis, and compares the proposed method with traditional transient protection.

### 4.1. Simulation System

**Figure 5:**
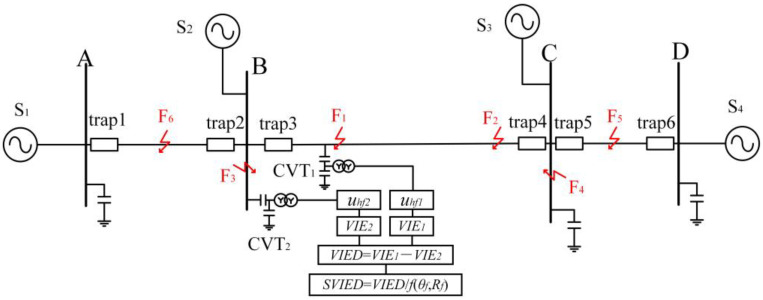
Simulation system.

The 500 kV UHV transmission simulation system established in ATP-Draw is utilized to validate the suggested protective strategy. The system model is depicted in Figure 5. The system is configured with a sample frequency of 400 kHz, and the protected objects are line BC and bus B. Traps are positioned at the start and end of each transmission line. The system’s equivalent power supplies, S_1_, S_2_, S_3_, and S_4_, are rated at 15 GVA, 6 GVA, 12 GVA, and 11 GVA, respectively. Voltage transformer CVT_1_ collects the voltage at the head end of line BC, and voltage transformer CVT_2_ collects the voltage at bus B. F_1_ represents a malfunction on line BC, located one kilometer from bus B. F_2_ represents a malfunction on line BC, located one kilometer from bus C. F_3_ represents a fault at bus B. F_4_ represents a fault at bus C. F_5_ represents a fault on line CD, located 1 km from bus C. F_6_ denotes a fault on line BA at 1 km from bus B. Simulation experiments were conducted to investigate various fault conditions with initial fault angles (0~360°) and transition resistances (0 Ω~250 Ω). Due to layout constraints, the following only lists the fault characteristics for transition resistances of 0 Ω, 10 Ω, 100 Ω, and 250 Ω and initial fault phase angles of 1°, 5°, 45°, and 90°.

### 4.2. Internal Faults

#### 4.2.1. Line BC Internal Fault

**Figure 6:**
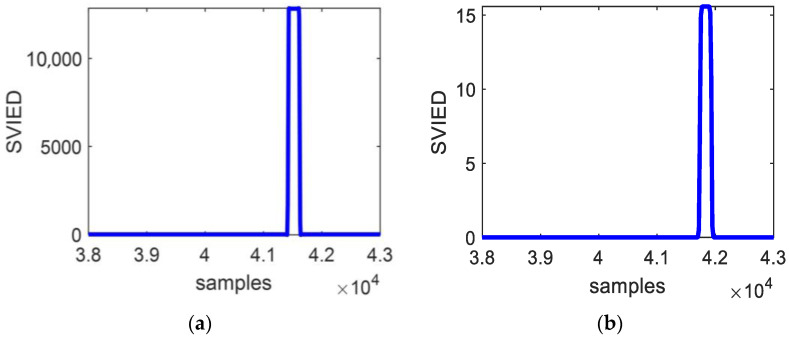
Line BC fault. (**a**) Fault at point F_1_; (**b**) fault at point F_2_.

Figure 6 shows the *SVIED* change diagram for the fault characteristic when the malfunction beginning phase angle is 45° and the malfunction impedance is 5 Ω, corresponding to a short-circuit grounding malfunction in phase A at points F_1_ and F_2_ on transmission line BC. The diagram clearly demonstrates that, upon fault occurrence, the *SVIED* increases sharply, making the fault characteristics highly apparent.

The values of the malfunction characteristic *SVIED* are shown in Table 2 for faults at points F_1_ and F_2_ under different fault condition attributes. The *SVIED* values are all positive real numbers for internal faults on line BC. In Table 1, the maximum multiplier between the *VIED* values is 869 under the condition of equal transition resistance and 3 under the same fault initial angle. In Table 2, the maximum multiplier between the *SVIED* values at the same fault location is 6 under the condition of equal transition resistance, and the maximum multiplier between the *SVIED* values is 1.1 under the same fault initial angle. From this, it can be clearly concluded that *VIED*, when fused with fault condition attributes, can effectively overcome the influence of fault attributes.

#### 4.2.2. Internal Bus B Fault

**Figure 7:**
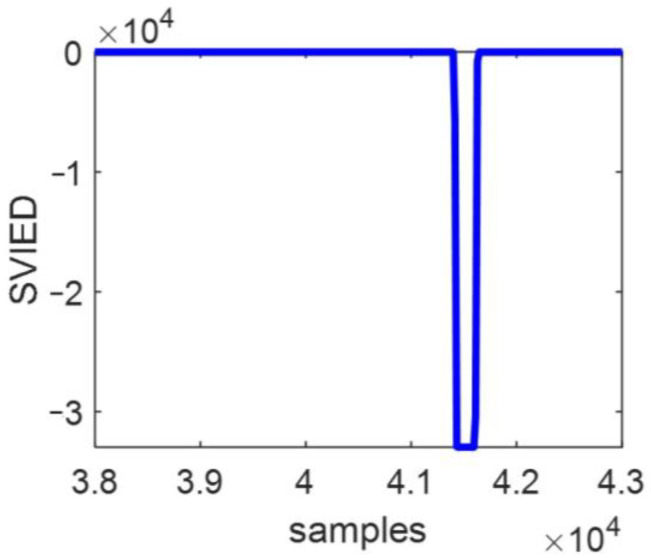
Bus B fault.

The *SVIED* for a malfunction impedance of 5 Ω when an A-phase ground short-circuit malfunction emerges at the busbar B with a malfunction beginning phase angle of fault of 45° is shown in Figure 7. After the fault occurs, the *SVIED* steeply and abruptly changes to a negative number, and its absolute value of *SVIED* becomes very large, making the fault characteristics clearly evident.

The fault characteristic *SVIED* values for different fault condition attributes at point F_3_ are shown in Table 3. For the bus B faults, the *SVIED* values are all negative real numbers. This is significantly different from the line fault data within the district, as shown in Table 2. Similarly, the data for the bus B fault differs by at least a factor of 169 from the data for the fault at point F_6_, which is located outside the zone in the same direction. This enables a clearer distinction between faults occurring at buses within the zone and those at other locations.

### 4.3. External Failure

**Figure 8:**
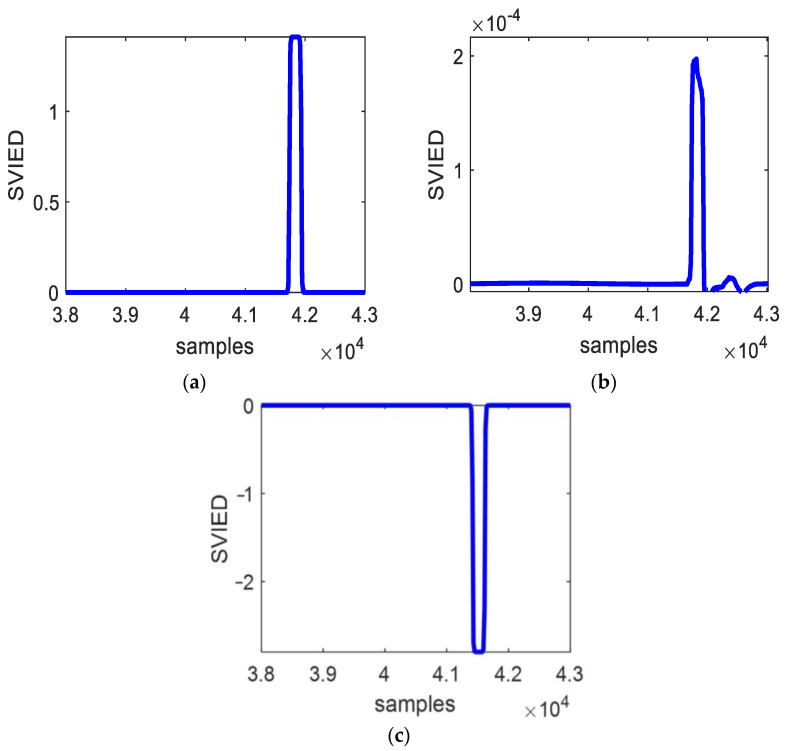
External fault. (**a**) Bus C (F_4_ point) fault; (**b**) fault at point F_5_ of line CD; (**c**) F_6_ fault of line BA.

The fault characteristic *SVIED* values for the A-phase ground short-circuit faults at point F_4_ on bus C, point F_5_ on line CD, and point F_6_ on line BA are shown in Figure 8. These faults are analyzed under a malfunction beginning phase angle of 45° and a malfunction impedance of 5 Ω. Figure 8 illustrates that the fault characteristic *SVIED* changes significantly when a malfunction occurs outside the protection zone. However, the information entropy difference is much smaller compared to when a malfunction emerges within the protection zone.

The *SVIED* values for different fault condition attributes are shown in Table 4. The data in Table 4 indicate that the *SVIED* is positive for the out-of-area faults at points F_4_ and F_5_ in the positive direction of line BC and negative for the out-of-area fault at point F_6_ in the opposite direction from line BC. Compared with the data in Table 2, the *SVIED* values at point faults F_4_ and F_5_ in the same direction are significantly different in amplitude, although they are the same in terms of positivity and negativity as those at point faults F_1_ and F_2_. The same is true for F_6_ and F_3_.

### 4.4. Failure Criteria Setting

From the analysis of the data in Table 2, Table 3 and Table 4, it is evident that for line BC faults within the protection zone, *SVIED* > 10.6; for bus B faults within the protection zone, *SVIED* < −3000; and for faults outside the protection zone, −7 < *SVIED* < 4. The combined fault characteristics for bus faults within the protection zone, line faults within the protection zone, and faults outside the protection zone show significant differences. Therefore, considering protection sensitivity and other requirements, the fault identification criteria can be adjusted as follows:

1. When *SVIED* > 6, it is identified as a line BC fault within the protected area.
2. When *SVIED* < −1500, it is judged to be a bus B fault within the protection area.
3. Otherwise, it is deemed to be a fault external to the protected area.

For reasons of space, only single-phase ground fault studies are listed above, and for other types of fault judgment studies, we have demonstrated that the proposed algorithm is equally effective.

### 4.5. Discussion

#### 4.5.1. Analysis of Influencing Factors

In addition to the factors mentioned in Section 3.3, the fault location also affects the fault characteristics. The farther the fault point is from the protection device, the smaller the fault feature detected by the protection device. However, this phenomenon does not follow a specific rule that can be used. In order to ensure the validity and accuracy of the research results, in the fault timing of the proposed method, the influence of fault location has been taken into account, that is, the fault is set at both the beginning and the end of the line. In the case of different fault locations, this method can still protect the whole length of the line.

#### 4.5.2. Quantitative Data Analysis

For the *VIED* data without information fusion with fault condition attributes, Table 1 shows that within the same fault location, the maximum difference in *VIED* under different working conditions is 869.23 times greater (*R_f_* = 250, *θ_f_* = 1° and 90°). After the fusion of fault characteristics and working condition attributes, the maximum difference in *VIED* at the same location (F_2_ fault) is reduced by 5.97 times (*R_f_* = 0, *θ_f_* = 1° and 90°). Additionally, in Table 2, Table 3 and Table 4, the difference in *VIED* under different working condition attributes at the same fault location reaches a value that is up to 36.14 times greater (F_5_ fault). At the same fault location, under different fault conditions, the fluctuation of *SVIED* after fault condition attribute fusion is greatly reduced, which enhances the reliability of protection.

When a line-end fault and a remote bus fault occur, the *VIED* without information fusion of fault condition attributes shows 38.9% cross-data overlap. To ensure that all internal faults on the line are detected, 36.1% of external faults would result in false operations. Conversely, to prevent false operations for external faults, the rejection rate for internal faults within an 11° range around the zero-crossing point is 6.1%. After the fusion of fault characteristics and condition attributes, all the data in Table 1 (remote fault point F_2_ in the protection zone) and Table 4 (remote bus fault point F_4_ outside the zone) show no intersection. This method effectively addresses the traditional challenges in transient protection, particularly with regard to terminal line faults and remote bus fault identification.

#### 4.5.3. Comparison with the Traditional Transient Protection Method

Table 5 compares the proposed protection method with existing transient protection techniques. The advantages of this approach include 100% protection of the line, protection of both the bus and the line, and immunity to the effects of *θ_f_* and *R_f_*.

## 5. Conclusions

In this paper, it is found that fault initial phase angle and transition resistance are two key factors affecting the sensitivity and reliability of transient protection in weak fault situations. By mining the equipment configuration information of the bus and line, as well as the frequency characteristics of the line borders, the difference in information entropy between the high-frequency components of bus voltage and those of line voltage is introduced. This difference is used to construct the malfunction instantaneous characteristics. The malfunction instantaneous feature is given two fault condition attributes: the malfunction beginning phase angle and malfunction impedance. This approach transforms the traditional two-dimensional fault transient feature, which only includes time attributes, into a four-dimensional fault transient feature that incorporates both time attributes and two fault condition attributes. By integrating the fault transient characteristics with the fault condition attributes, a comprehensive fault transient characteristic containing fault attribute information is obtained. This helps eliminate the differences in fault transient characteristics caused by fault engineering attributes to the maximum extent, overcomes the adverse effects of fault attributes on transient protection, and thereby improves protection reliability. Additionally, it can also protect a line and bus at the same time. A series of ATP-Draw simulation experiments verify the accuracy and reliability of the proposed algorithm. However, the proposed algorithm still has limitations: the method is only applicable to transmission systems with traps installed, and the accuracy of the algorithm depends on the accurate calculation of *θ_f_* and *R_f_*. To further improve the reliability of protection, future research should focus on developing a faster and more accurate online method for calculating *R_f_*.

## Figures and Tables

**Table 1:** *VIED* under different fault condition attributes.

*θ_f_* [°]	*R_f_* [Ω]			
	0	5	100	250
F_1_ fault (1 km from bus N)				
1	0.0213	0.0191	0.0089	0.0039
5	0.3164	0.2855	0.1375	0.0625
45	7.4437	6.9260	3.9524	2.0139
85	10.6036	10.0547	6.2759	3.3713
90	10.6400	10.0923	6.3082	3.3912

**Table 2:** *SVIED* of line BC fault with different fault condition attributes.

*θ_f_* [°]	*R _f_* [Ω]			
	0	10	100	250
F_1_ fault				
1	14,223	14,581	17,684	20,644
5	12,241	12,688	9704	9096
45	13,385	12,858	9732	6283
90	16,002	15,453	12,293	8779
F_2_ fault				
1	63.5396	63.8713	68.8477	72.6180
5	41.9314	42.3668	47.1650	51.8749
45	14.9545	15.5814	20.5559	25.3457
90	10.6400	11.3013	16.3304	21.2439

**Table 3:** *SVIED* of bus B fault with different fault condition attributes.

*θ_f_* [°]	*R_f_* [Ω]			
	0	10	100	250
1	−21,459	−21,210	−3187	−3097
5	−25,228	−23,132	−3557	−3242
45	−37,954	−32,968	−3923	−3330
90	−43,589	−38,127	−5141	−1234

**Table 4:** *SVIED* of outer zone fault with different fault condition attributes.

*θ_f_* [°]	*R_f_* [Ω]			
	0	10	100	250
F_4_ fault				
1	3.8865	3.3777	0.9414	0.5170
5	2.8677	2.5309	0.7496	0.4261
45	1.5688	1.4107	0.4786	0.2893
90	1.3654	1.2364	0.4365	0.2682
F_5_ fault				
1	0.00243	0.00251	0.00376	0.00759
5	0.00017	0.00013	0.00029	0.00035
45	0.00022	0.00027	0.00025	0.00026
90	0.00020	0.00020	0.00028	0.00021
F_6_ fault				
1	−6.5924	−6.6109	−6.7631	−7.3047
5	−5.2036	−5.3444	−5.0476	−5.2726
45	−2.6976	−2.7996	−3.6653	−4.5142
90	−1.9865	−2.0547	−2.6578	−3.2635

**Table 5:** Comparison with the traditional transient protection method.

Ref.	Scope of Protection	Required Information	Influenced by *θ_f_* and *R_f_*	Distinguish Line End Fault from Remote Bus Fault	The Condition of 100% Protection of the Protected Object
[8]	A Bus	Electrical signals of all lines connected to the bus	√	N	*θ_min_* = 5°
[19]	A line	Single-ended signals for lines	√	×	*θ_min_* > 5°
[20]	A line	Single-ended signals for lines	√	×	*θ_min_* = 5°; R_max_ = 300; 220 kV.
[26]	A line	Single-ended signals for lines	√	×	62.5% of the total length of the line; The line to install the trap.
This paper	A bus and a line	Single-ended signals for lines	×	√	The line to install the trap

Note: N indicates not studied or reported.

## Data Availability

The data presented in this study are available on request from the corresponding author.
